# An Account of the Practice of One of the Physicians of the Westminster General Dispensary, and of the Western Dispensary, from the 20th of April, to the 20th of May, 1807

**Published:** 1807-06

**Authors:** Samuel Fothergill

**Affiliations:** Southampton Street, Strand


### *An Account of the Practice of one of the Physicians of the Westminster General Dispensary, and of the Western Dispensary, from the 20th of April, to the 20th of May,* 1807.

| | | | |
|---|---|---|---|
| ACUTE DISEASES. | | Colic - - - | 1 |
| Typhus - - - | 4 | Dyspepsia - - | 5 |
| Scarlatina - - | 1 | Bilious Vomiting - | 4 |
| Pleurisy - - - | 2 | Gout - - - | 2 |
| Peripneumony - - | 4 | Diarrhœa - - - | 4 |
| Catarrh - - - | 3 | Dysentery - - | 1 |
| Hæmoptysis - - | 2 | Dropsy - - - | 4 |
| Acute Rheumatism - | 5 | Jaundice - - - | 2 |
| Enteritis - - - | 1 | Pyrosis - - - | 2 |
| Acute Diseases of Infants | 8 | Dysuria - - - | 1 |
| CHRONIC DISEASES. | | Asthenia - - - | 8 |
| Cough and Dyspnœa - | 30 | Paralysis - - - | 3 |
| Asthma - - - | 2 | Cephalalgia and Vertigo | 5 |
| Pulmonary Consumption | 4 | Epilepsy - - - | 1 |
| Pleurodyne - - | 3 | Worms - - - | 3 |
| Chronic Rheumatism | 8 | Cutaneous Diseases - | 6 |
| Lumbago - - - | 3 | Amenorrhœa - - | 3 |
| Gastrodynia - - | 4 | Chlorosis - - - | 1 |
| Enterodynia - - | 2 | Dysmenorrhœa - | 1 |
| Epistaxis - - - | 1 | Menorrhagia - - | 2 |
| Hæmatemesis - - | 2 | Leucorrhœa - - | 3 |

In a former Report, I stated the good effects of Aqua Ammoniæ Puræ, in the cure of Tic Douloureux. I have now considerable satisfaction, in being enabled to record another instance in which that painful malady yielded to a similar practice; adopted in consequence of the observations which I then made. The patient was the wife of a medical gentleman residing in Westminster; she had tried all the remedies usually recommended, but experienced no no relief till she took the aqua ammoniæ puræ. The particulars of the case I cannot at present communicate; but from the respectability of the party, I have no doubt the symptoms of the disease were accurately discriminated, and the result faithfully related.

With the mild agreeable weather which we have lately experienced, the general state of disease has become more favourable; winter complaints begin to disappear, whilst some of those, which in spring are often severe, at present very seldom occur.

Three of the cases of peripneumony were in the children of one family, of which the youngest, an infant, died; and the other two, though out of danger, are extremely weak.

In affections of the head, it is sometimes difficult to ascertain the remote, or predisposing cause; the symptoms may be occasioned by some organic disease, and they may be produced by intense application of mind, or by despondency; whilst the immediate exciting cause in either case, may be, an effusion of serum in the ventricles; inflammation of the membranes, forming coagulable lymph and pus; or some change in the brain itself; this however, can only be determined by observations made from morbid anatomy, and it is much to be regretted, that the prejudices of people should still impede the cause of medical science, by preventing the inspection of bodies after death. I have lately attended three cases, in which I had reason to suspect the brain was primarily affected. Two of them have already terminated fatally, and one affords some hope of recovery. The first of these patients was a respectable tradesman; he informed me, that about two weeks before I saw him, he had been affected with a severe cold, from which, however, he was now recovering; but wished to have something to procure sleep, his nights being extremely restless. I found him very feeble; he had scarcely slept for a week, and had no inclination for food. There was a peculiarly vacant expression of countenance, which sometimes appeared rather wild, and then changed to laughing. The bowels were regular; the urine rather scanty, clear, and of a reddish colour; the pulse regular and full, beating from 120 to 140 strokes in a minute: the skin was dry, the heat natural, and the tongue covered with a thick white fur. Upon drinking any liquid, he had a sensation as if the fluid was flowing from his mouth, instead of passing into it, so that he made considerable efforts whenever he drank; this continued to the last.—

Opium,

Opium, calomel, and digitalis, with purgatives, at first afforded some relief: the head was shaved and blistered, and cupping repeated; he took scarcely any nutriment, yet was not at all reduced by the bleeding; his pulse continuing remarkably firm. A gentle perspiration was excited, and after the third day, the opiate not occasioning sleep, was omitted.—Low delirium with confusion of thought were frequently observed. The pupil of the eye was slightly dilated. These symptoms gradually increased for about ten days, when he sank under his disease. Till the last day but one, he knew his friends, and, with some time to recollect himself, answered questions rationally; but throughout his illness, was in a state of extreme despondency respecting his affairs, which, however, I could not find were in a bad state, and therefore attributed his anxiety on that head to be the effect of his complaint.

SAMUEL FOTHERGILL.

*Southampton Street, Strand, May* 22, 1807.

